# Exosome-based detection of *EGFR* T790M in plasma and pleural fluid of prospectively enrolled non-small cell lung cancer patients after first-line tyrosine kinase inhibitor therapy

**DOI:** 10.1186/s12935-021-01761-x

**Published:** 2021-01-12

**Authors:** Yoonjung Kim, Saeam Shin, Kyung-A Lee

**Affiliations:** Department of Laboratory Medicine, Yonsei University College of Medicine, Seoul, Republic of Korea

**Keywords:** Liquid biopsy, Extracellular vesicles, Circulating tumor DNA, Non-small cell lung cancer, Epidermal growth factor receptor, Tyrosine kinase inhibitors

## Abstract

**Background:**

The exosomal nucleic acid (exoNA) from the plasma and pleural fluid can potentially provide means to identify genomic changes in non-small cell lung cancer (NSCLC) patients who develop resistance to targeted epidermal growth factor receptor (EGFR) inhibitor therapy.

**Methods:**

We compared the performance of the following tools to detect *EGFR* mutations in 54 plasma samples and 13 pleural fluid using cfDNA, combined TNA (exoTNA + cfTNA), or total cellular DNA: droplet digital PCR (ddPCR), the Cobas® *EGFR* Mutation Test v2 (Cobas) and NGS with Oncomine Pan-Cancer Cell-Free Assay.

**Results:**

All three of these platforms demonstrated 100% specificity in the detection of *EGFR* mutations in the plasma. In the detection of an activating mutation (exon 19 deletion and L858R), Cobas using cfDNA, ddPCR using combined TNA, and NGS using combined TNA showed a sensitivity of 93, 95.3, and 93.8%, respectively. For T790M mutation detection, the Cobas, ddPCR, and NGS showed a sensitivity of 64.7, 88.2, and 93.3%, respectively. Pleural fluid analysis revealed enrichment of the T790M mutant copies in the exosomes. ddPCR using exoTNA showed higher sensitivity than did total cellular DNA from the pleural fluid.

**Conclusion:**

These results demonstrated that combined TNA in the plasma and exoTNA in the pleural fluid can be used to evaluate low-abundant *EGFR* mutant copies in NSCLC.

## Background

The discovery of driver mutations in the epidermal growth factor receptor (*EGFR*) gene has led to a dramatic paradigm shift in the therapeutic strategies for advanced non-small-cell lung cancer (NSCLC). First- and second-generation tyrosine kinase inhibitors (TKIs), such as gefitinib, erlotinib, and afatinib, are standard therapies for patients with advanced NSCLC harboring mutations in *EGFR*. These mutations include deletion mutations in exon 19 and the L858R mutation in exon 21 [1, 2]. Unfortunately, acquired resistance to TKIs frequently develops within 1 or 2 years of initiating therapy. The most common mechanism of resistance, which accounts for ~ 60% of cases, is the acquired mutation T790M. *MET* amplification (5–7%) and *ERBB2* amplification (5–13%) occur less frequently than do the T790M mutation [3–5].

Most commonly, a cancer’s molecular profile is assessed using the tumor tissue. Longitudinal surveillance of clonal evolution, including acquired resistance alterations following TKI therapy, is essential for precision medicine. This frequent surveillance cannot be effectively achieved using tissue biopsy specimens, however, due to the possibility of inappropriate or inadequate sampling that misses portions of the tumor that are developing treatment resistance or have acquired new driver mutations due to tumor heterogeneity [6]. Therefore, liquid biopsy samples are considered an alternative means of detecting clinically relevant mutations in NSCLC patients undergoing TKI therapy [7].

Circulating tumor DNA (ctDNA) is the most commonly analyzed DNA to detect *EGFR* mutations in NSCLC [8]. Many technical platforms targeting ctDNA are already being implemented in clinical practice. However, the low abundance of ctDNA is an obstacle for detecting *EGFR* mutations in NSCLC patients with acquired TKI-resistance. In particular, patients with a low T790M copy number (< 10 copies/mL) have similar responses to osimertinib than do those with a higher T790M copy number (≥ 10 copies/mL) [9]. In one study, there were more patients with low T790M copy numbers than there were with higher T790M copy numbers [10]. Therefore, the pre-analytical steps for maximizing the tumor-derived nucleic acid concentration and ultra-sensitive analytical techniques were essentially required to achieve the optimal sensitivity of the *EGFR* mutation.

Exosomal nucleic acid (exoNA) has been studied as a target for cancer mutation testing. It has been shown to improve the sensitivity of detection in patients with limited T790M mutant copies of cell-free DNA (cfDNA), such as those with early-stage NSCLC or intrathoracic disease (M0/M1a) [7, 8, 11, 12]. Furthermore, body fluids such as bronchoalveolar lavage (BAL) and pleural fluids also have been tested to assess the feasibility of detecting *EGFR* mutations. These fluids have also been evaluated to determine which component (exosome, supernatant, and cell pellet) contained abundant tumor-derived nucleic acids in NSCLC patients [13, 14].

There is a constant need to improve the detection capability of clinically significant, but rare mutant alleles in clinical samples. Therefore, we compared the performance of different techniques (allele-specific real-time PCR, droplet digital PCR, and next-generation sequencing) using various sources of tumor-derived nucleic acids (cfDNA and exosome from plasma, exosome and total cellular DNA from pleural fluid). We demonstrated the optimal analytical method to improve the sensitivity of detecting *EGFR* mutations in different types of clinical samples.

## Materials and methods

### Patients and clinical specimens

Patients were included in the two following criteria were all met: (1) they had a diagnosis of NSCLC and confirmed *EGFR* mutation on tissue genotyping, and (2) showed disease progression on first- or second-generation EGFR-TKI. Between November 2017 and September 2019, 64 consecutive NSCLC patients were prospectively enrolled. The *EGFR* genotyping result of tissue (*n* = 61) was obtained from specimens at initial diagnosis (*n* = 58) or from specimens matched with plasma (*n* = 3). Patients consented to the protocol approved by the Institutional Review Board of Gangnam Severance Hospital and Kangnam Sacred Heart Hospital. Fifty-four whole blood samples were obtained from 54 patients, and 13 pleural fluids was obtained from 13 patients. Three pleural fluid and blood samples were collected at the same time in three patients (P23, P25, and P54).

### Preparation of nucleic acid from plasma and cDNA synthesis

The blood samples were collected in cell-free DNA Blood Collection Tubes (Roche, Pleasanton, CA, USA). Plasma isolation was performed using a two-step plasma separation procedure. The blood samples were centrifuged at 1200* g* for 10 min, followed by high-speed centrifugation at 16,000* g* for 10 min. The plasma aliquots were stored at − 80 °C [15].

Exosomes were isolated from the plasma using ExoQuick™ (System Biosciences, Mountain View, CA, USA). We extracted the plasma cfDNA and exosomal total nucleic acid (exoTNA) using the MagMAX™ Cell-Free DNA Isolation Kit (Thermo Fisher Scientific, Waltham, MA, USA) and MagMAX™ Total Nucleic Acid Isolation Kit (Thermo Fisher Scientific), respectively [11]. The concentrations of cfDNA and exoTNA were assessed with the Qubit™ 3.0 Fluorometer using the Qubit™ dsDNA HS Assay Kit (Thermo Fisher Scientific). cDNA synthesis was performed using a SuperScript™ VILO™ cDNA Synthesis Kit (Invitrogen, Carlsbad, CA, USA).

### Preparation of nucleic acid from exosomes, and cell pellets and supernatants from pleural fluid

The pleural fluid was centrifuged at 3000* g* for 15 min to remove cellular debris. The exosomes were isolated from the supernatants using ExoQuick™. A nucleic acid of exosomes was extracted with the MagMAX™ Total Nucleic Acid Isolation Kit. cDNA synthesis was performed using a SuperScript™ VILO™ cDNA Synthesis Kit. We extracted the total DNA from the cell pellet using the QIAamp DNA Mini Kit (QIAGEN, Hilden, Germany). The sizes of the DNA fragments in the exosomes were assessed using a 2200 TapeStation Instrument (Agilent Technologies, Santa Clara, CA, USA) with the Genomic DNA ScreenTape System. The DNA concentration was assessed with the Qubit 3.0 Fluorometer (Thermo Fisher Scientific). The RNA yield and size distribution were analyzed using an Agilent 2100 Bioanalyzer with an RNA 6000 Pico kit (Agilent Technologies, Foster City, CA, USA).

### Cobas *EGFR* Mutation Test v2

For the Cobas *EGFR* assay, 75 μL of DNA from each plasma sample (2 mL) was loaded into three reaction wells (25 μL DNA per well). Amplification and detection were performed using the Cobas z 480 analyzer (Roche Molecular Systems, Inc., Branchburg, NJ, USA). The data were interpreted using the Cobas z 480 software if the positive and negative controls showed valid results.

### Droplet digital PCR (ddPCR)

The ddPCR assays were performed using the PrimePCR™ ddPCR™ Mutation Detection Assay kit, and the PrimePCR™ ddPCR™ *EGFR* Exon 19 Deletions Screening Kit (Bio-Rad Laboratories, Hercules, CA, USA) [11]. Tumor-derived nucleic acids (cfDNA and exoTNA) were extracted from 1 to 2 mL of plasma or pleural fluid. The ddPCR assay for detecting *EGFR* mutations was validated using the Multiplex I cfDNA Reference Standards (Horizon Discovery, Cambridge, UK) and healthy control samples from a previous study [11]. Briefly, the amplifications were performed in a reaction volume of 20 μL on a QX100 Droplet Digital PCR System (Bio-Rad). The 20 μL of the PCR mixture was composed of 10 μL Bio-Rad Super mix TaqMan, 1–2 μL of each amplification primer/probe mix, and 8–9 μL of nucleic acids (NAs). Thermal cycling comprised an initial denaturing and polymerase hot-start activating step of 10 min at 95 °C, followed by 40 cycles of 95 °C for 30 s and 55 °C for 60 s. The results were analyzed with QuantaSoft v.1.7.2 software (Bio-Rad) and reported as copies per milliliter of plasma. The ddPCR assay was validated for detecting *EGFR* mutations and determining the limit of detection (LOD) in a previous study [11]. The assays were considered “positive” if the measured event rate was ≥ 2 events/assay and “negative” if the event rate within a gated region was < 2 events/assay.

### Next-generation sequencing (NGS)

For NGS, a library was prepared using the Oncomine Pan-Cancer Cell-Free Assay (Thermo Fisher Scientific) targeting 52 cancer-associated genes (Additional file 1: Table S1). The NGS panel is designed to detect single nucleotide variations, small indels, copy-number alterations, and gene fusions. The libraries were prepared using > 5 ng nucleic acid input following the Oncomine Pan-Cancer Cell-Free Assay user guide. Templating and sequencing were performed using the Ion 540™ Kit on the Ion Chef™ and on the Ion S5 XL system (Thermo Fisher Scientific). Alignment to the hg19 human reference genome and variant calling were performed using the Torrent Suite™ and Ion Reporter™ software version 5.10, respectively. The Torrent Suite™ Software provided molecular coverage depth and read coverage depth at the target base; therefore, this software was able to increase the detection sensitivity for low-frequency variants [16, 17].

The average of median read coverage and median molecular coverage were 36,095 × and 1934  ×, respectively. Variants with an allele frequency of > 0.1% were reported. The measured allele frequency (%) was calculated as the mutant coverage depth divided by the total coverage depth.

### Statistical analysis

The statistical analysis was performed using R (version 3.5.2, http://www.r-project.org) and MedCalc software (https://www.medcalc.org/). The nonparametric data were analyzed using the Kruskal–Wallis tests. Multiple comparisons were made using Dunn's test. A *p*-value < 0.05 was considered statistically significant.

## Results

### Patient and tumor characteristics

The baseline clinical and pathological characteristics of the 64 patients are described in Table 1. More women in our patient cohort than men (73.4% vs. 26.6%) might be reflected by the differential distribution of *EGFR* mutation between male and female patients with NSCLC, as reported by previous studies [18, 19]. Most patients had stage IV disease. Only two patients had M0 stage disease. Two patients had T790M mutations in the tissue from a repeat biopsy sample. All of the patients received gefitinib, erlotinib, and/or afatinib as first line treatment of their advanced or metastatic disease.

**Table 1:** Patient characteristics

Clinical characteristics	Total 64 patients, *n* (%)
Age, median (range), years	66 (40–85)
Sex	
Female	47 (73.4)
Male	17 (26.6)
Histologic type	
Adenocarcinoma	62 (96.9)
Squamous cell carcinoma	2 (3.1)
Tumor stage	
IIa	1 (1.9)
IIIb	1 (1.9)
IVa	36 (66.7)
IVb	26 (48.1)
M category^a^	
M0	2 (3.1)
M1a	24 (37.5)
M1b	15 (23.4)
M1c	23 (35.9)
Tissue *EGFR* genotyping^b^	
T790M and exon 19 deletion^c^	2 (3.1)
Exon 19 deletion	33 (51.6)
L858R	23 (35.9)
Other^d^	6 (9.4)
Prior treatment	
Gefitinib	34 (53.1)
Erlotinib	11 (17.2)
Afatinib	16 (25.0)
Afatinib + Gefitinib	2 (3.1)
Erlotinib + Gefitinib	1 (1.6)

^a^According to the 8th TMN edition, M1a indicates lung metastases or pleural/pericardial malignant effusion or nodules; M1b indicates a single metastatic lesion in a single distant organ; M1c indicates multiple lesions in a single organ or multiple lesions in multiple organs

^b^Tissue genotyping was performed using the PANAMutyper *EGFR* kit (PANAGENE Inc., Daejeon, Korea)

^c^Two patients had T790M mutations that were identified though re-biopsy after EGFR-TKI therapy

^d^One exon 20 insertion, one L861Q, one G719X and S768I, and three patients with no tissue genotyping result

*Other* not otherwise specified, *TKI* tyrosine kinase inhibitor

At the time of progression, patients were tested for both the persistence of initial activating mutations (exon 19 deletion and L858R) and the onset of T790M resistance mutations using the Cobas assay in the plasma cfDNA. This testing was performed to determine the patients’ eligibility for osimertinib use. The Cobas assay showed the persistence of initial activating mutations in 41 patients. Among them, T790M resistance mutations were identified in 11 patients (11/41, 26.8%). Fourteen patients had negative results for both activating and resistance mutations.

### Quantification of exoTNA and cfDNA in clinical samples

The cfDNA used in the Cobas® *EGFR* Mutation Test were extracted from plasma samples (2 mL). The exoTNA and cfDNA for the ddPCR and NGS were extracted from equal volumes of plasma samples from 54 patients. Among them, three samples were not available for NA quantification due to a lack of volume. The cfDNA amount for the Cobas® *EGFR* Mutation Test and both exoNA and cfDNA amount for the ddPCR assay are depicted in (Additional file 1: Fig. S1). The median amount level of cfDNA for the Cobas® *EGFR* Mutation Test was 16.8 ng. Those of cfDNA and exoNA for ddPCR were 10.3 ng and 11.41 ng, respectively. There was a significant difference between the NA yield of three groups (Kruskal–Wallis test, *p* = 0.0002). There were also significant differences in the NA yield between the cfDNA for Cobas® *EGFR* Mutation Test and ExoNA, and the cfDNA for the ddPCR assay (*p* < 0.05, Dunn's multiple comparisons test).

### Comparison among Cobas, ddPCR and NGS using plasma from NSCLC patients who progressed under treatment with an EGFR-TKI

We compared the diagnostic performance of Cobas (cfDNA), ddPCR (cfDNA + exoTNA), and NGS (cfDNA + exoTNA) to detect an *EGFR* activating mutation (exon 19 deletion and L858R, Table 2) and the resistance T790M mutation (Table 3). NGS was possible in 38 of 54 cases with sufficient plasma samples. There were two patients who were confirmed to have T790M mutations on repeat tissue biopsy (P19 and P40, Additional file 1: Table S2). The plasma analysis of one patient (P19) revealed positive results from all three platforms (Cobas, ddPCR, and NGS). In contrast, plasma analysis in another patient (P40) showed negative results in all three platforms. Although most patients did not undergo tissue re-biopsy, all cases with T790M positivity detected from one or more liquid biopsy platforms (Cobas, ddPCR, or NGS) had disease progression after > 10 months of TKI treatment. Therefore, T790M positivity was defined as “true positive,” even if it was detected in only one liquid biopsy platform. An activating mutation was considered a “true positive” if there was positive detection from two or more liquid biopsy platforms, or positive detection from tissue genotyping and one or more liquid biopsy platforms. The sensitivities of Cobas, ddPCR, and NGS for detection activating mutations in the plasma were 93% (95% confidence interval, 91.7–94.3), 95.3% (94–96.6), and 93.8 (92.2–95.4), respectively (Table 2). Among three samples (P29, P40, P54) with false-negative activating mutations according to the Cobas assay, exon 19 deletion mutations were detected in two samples. These included one mutation from ddPCR (P54, 2 positive droplets/829 total droplets) and another from NGS (P29, variant allele frequency of 0.4%). The NGS assay failed to detect exon 19 deletion mutations in two samples (P20, P40). Only one exon 19 deletion of P20 was detected using both Cobas and ddPCR (6 mutant droplets) techniques.

**Table 2:** Comparing the detection of the *EGFR* activating mutation between the Cobas assay, ddPCR and NGS using plasma from NSCLC patients

*EGFR* genotype	TP and TN^a^ (38 cases with NGS results)	Cobas assay using cfDNA (*n* = 54)		ddPCR using cfDNA + exoTNA (*n* = 54)		NGS using cfDNA + exoTNA (*n* = 38)	
		Mutant type	Wild-type	Mutant type	Wild-type	Mutant type	Wild-type
Mutant type	43 (32)	40	3	41	2	30	2
Wild-type	11 (6)	0	11	0	11	0	6
Sensitivity,% (95% CI)		93 (91.7–94.3)		95.3 (94–96.6)		93.8 (92.2–95.4)	
Specificity, % (95% CI)		100 (98.7–101)		100 (98.7–101)		100 (98.4–102)	
Accuracy, % (95% CI)		94.4 (93.1–95.7)		96.3 (95–97.6)		94.7 (93.1–96.3)	

^a^‘True positive’ was defined as a activating mutation positive in more than two or more liquid biopsy platforms among Cobas, ddPCR, and NGS or positive in tissue genotyping and one or more liquid biopsy platforms. ‘True negative’ was defined as activating mutation negative in all tested liquid biopsy platforms

*NSCLC* non-small cell lung cancer, *TP* true positive, *TN* true negative, *CI* confidence interval

**Table 3:** Performance comparison for detection of the *EGFR* T790M mutation between the Cobas assay, ddPCR, and NGS using plasma from NSCLC patients

*EGFR* genotype	TP and TN^a^ (38 cases with NGS results)	Cobas assay using cfDNA (*n* = 54)		ddPCR using cfDNA + exoTNA (*n* = 54)		NGS using cfDNA + exoTNA (*n* = 38)	
		Mutant type	Wild-type	Mutant type	Wild-type	Mutant type	Wild-type
Mutant type	17 (15)	11	6	15	2	14	1
Wild-type	37 (23)	0	37	0	37	0	23
Sensitivity,% (95% CI)		64.7 (63.4–66)		88.2 (86.9–89.5)		93.3 (91.7–94.9)	
Specificity, % (95% CI)		100 (98.7–101)		100 (98.7–101)		100 (98.4–102)	
Accuracy, % (95% CI)		88.9 (87.6–90.2)		96.3 (95–97.6)		97.4 (95.8–99)	

^a^‘True positive’ was defined as a positive T790M mutation in one or more liquid biopsy platforms among Cobas, ddPCR, and NGS; ‘True negative’ was defined as T790M mutation negative in all tested liquid biopsy platforms

*NSCLC* non-small cell lung cancer, *TP* true positive, *TN* true negative, *CI* confidence interval

The sensitivities of Cobas, ddPCR, and NGS to detect T790M mutations in plasma were 64.7% (95% confidence interval, 63.4–66), 88.2% (86.9–89.5), and 93.3 (91.7–94.9), respectively (Table 3). Among 17 patients with T790M mutations that were confirmed by tissue genotyping and on one additional liquid biopsy platform, the Cobas assay did not detect the T790M mutation in six patients, although it identified coexisting activating mutations in five patients. These false negative T790M cases from the Cobas assay consisted of cases with very few copies of T790M in the plasma according to the results of the ddPCR (3–6 mutant droplets) and NGS assays (0.4–1.2% variant allele frequency). However, one case (P40) in which T790M was only identified on tissue re-biopsy was excluded (Additional file 1: Table S2). In addition, the detection rates of T790M with ddPCR (cfDNA + exoTNA) or NGS (cfDNA + exoTNA) were as much as 20–30% higher than were those using Cobas (cfDNA) (Table 3).

The *EGFR* T790M mutation is the most frequent mutation associated with the development of acquired resistance to EGFR-TKIs. The T790M was detected in the plasma of 17 patients (31.5%) who progressed despite treatment with an EGFR-TKI (*n* = 54). The T790M positivity rate was 27.8% using ddPCR (15/54), but only 20.4% using Cobas (11/54). The genetic alterations related to EGFR-TKI tolerance were further explored using NGS assay. Seven patients (7/38, 18.4%) were found to have resistance mutations other than *EGFR* T790M. *KRAS* mutations (G12D and G13D) were identified in two patients (P28 and P39), and *PIK3CA* mutations (E545K) were found in three patients (P30, P33, and P37). Gene amplifications that are associated with TKI resistance were identified in two patients (*EGFR* amplification in P8 and *EGFR/CDK4* amplifications in P52).

Furthermore, six patients (6/38, 15.8%) harbored various copies of T790M mutations (2–2098 positive droplets in ddPCR) along with other genetic mutations that are known to be associated with TKI resistance (including *CDK6/KRAS/CCND1* amplifications in P19, *PIK3CA* in P20 and P43, *EGFR/CDK6* amplifications in P22, *EGFR* amplification in P23, and *BRAF* in P50).

### Assessment of T790M mutant alleles according to different sources of tumor-derived nucleic acids from pleural fluid

Three pleural fluid samples were used to analyze different sources of tumor-derived nucleic acids (exosomes, cell pellets, and supernatants) and determine which compartment was suitable for the analysis. The event numbers of T790M positive and wild type cases are depicted in (Additional file 1: Fig. S2). Analysis of three samples revealed the largest number of mutant copies in exoTNA, followed by exoDNA and total DNA from the supernatants. In one sample (Sample 2), the total DNA from the cell pellet demonstrated the largest number of mutant copies compared to that in the exoTNA, exoDNA, and total DNA from the supernatants. Therefore, we selected the exoTNA, total cellular DNA and tested the pleural fluid samples from 13 NSCLC patients with disease progression after EGFR-TKI treatment (Additional file 1: Table S3). The ddPCR, using the total DNA extracted from the cell pellet, revealed T790M mutations in 38.5% of tested pleural fluid samples (5/13) (Table 4). Using the exoTNA extracted from the supernatant, two more cases (P56 and P61) also demonstrated T790M positivity (53.8%, 7/13). Two samples showed very low mutant copies in ddPCR (including four positive droplets among 4595 total droplets, and two positive droplets among 3900 total droplets). Therefore, the ddPCR using the total cellular DNA showed 71.4% sensitivity compared with the exoTNA from the supernatant.

**Table 4:** Performance comparison for the detection of the *EGFR* T790M mutation between exoTNA from supernatant and total DNA from cell pellets isolated from pleural fluid (*n* = 13)

*EGFR* genotype	TP and TN^a^	ddPCR using exoTNA extracted from supernatants		ddPCR using total DNA extracted from cell pellet	
		Mutant type	Wild-type	Mutant type	Wild-type
Mutant type	7	7	0	5	2
Wild-type	6	0	6	0	6
Sensitivity,% (95% CI)		100 (97.3–103)		71.4 (68.7–74.1)	
Specificity, % (95% CI)		100 (97.3–103)		100 (97.3–103)	
Accuracy, % (95% CI)		100 (97.3–103)		84.6 (81.9–87.3)	

^a^‘True positive’ was defined by a positive T790M mutation in one or more liquid biopsy platforms among exoTNA from supernatants and cellular total DNA from pleural fluid; ‘True negative’ was defined as T790M mutation negative in all tested liquid biopsy platforms

*TP* true positive, *TN* true negative, *CI* confidence interval

The distribution of isolated nucleic acids is shown in (Additional file 1: Fig. S3). The high molecular weight DNA (10–15 kb long) was more abundant in exosomes from pleural fluids than it was from other samples.

## Discussion

In this study, we assessed the performance of three liquid biopsy platforms (including the Cobas assay, ddPCR, and NGS) on different sources of tumor-derived nucleic acids (cfDNA, exoTNA, and total DNA) and clinical specimens (plasma and pleural fluid). The plasma analysis indicated that a combination of cfDNA and exoTNA showed higher sensitivity than that did exoTNA or cfDNA alone. In the pleural fluid analysis, exoTNA from supernatants showed enrichment of the T790M mutant copies by other sources of nucleic acids from supernatants or cell pellets.

The advantage of ddPCR is that it can sensitively detect low-abundant target molecules. Therefore, ddPCR is often used for ctDNA testing [20]. Currently, most liquid biopsy methods used in clinical research and practice only used cfDNA to detect cancer mutations. Even with ddPCR, which is currently considered the most sensitive technique, the sensitivity for detecting *EGFR* T790M mutations is only 70–77% of that when using tissue testing if cfDNA is used alone [20, 21]. Therefore, it is crucial to increase the sensitivity of the liquid biopsy technique to detect resistant mutations and identify patients who could potentially benefit from osimertinib therapy. In this study, we found that the sensitivity of ddPCR also depends on the type of specimens and the sources of nucleic acid. The use of combined TNA (cfDNA + exoTNA) in the plasma and exoTNA in the pleural fluid allowed for the detection of target mutations more sensitively than that using cfDNA or total DNA alone.

Both ddPCR and real-time PCR (Cobas) are sensitive and relatively easy and fast tests. However, only targeted hot-spot mutations are detectable using these techniques. The advantage of NGS, in contrast, is that many panel genes can be examined at once to detect known resistance mutations of EGFR-TKI other than *EGFR* hot-spot mutations. For instance, *KRAS, EGFR,* and *PIK3CA* mutations are known to be acquired resistance mechanisms to EGFR-TKIs [22, 23]. Amplifications in *KRAS*, *EGFR*, and cell cycle genes (including *CCND1*, *CDK4*, and *CDK6*) have also been reported as TKI resistance mechanisms [24, 25]. In this study, we detected mutations in *KRAS* and *PIK3CA,* and amplifications in *EGFR* and *CDK4* in patients who did not have the *EGFR* T790M mutation.

In addition, some patients had other resistance mutations that were found in addition to *EGFR* T790M. The phenomenon by which T790M and other resistant mutations occur simultaneously is clonal heterogeneity [26]. For instance, the *BRAF* and *PIK3CA* mutations and *EGFR* amplification can coexist with T790M in patients who progressed despite EGFR-TKI therapy [27–29]. The clinical significance of this co-occurring mutation is that it can affect the treatment outcomes [26].

The size distribution of tumor-derived nucleic acids in the exosome may be a critical consideration for selecting an extraction method. Our group previously showed that a short-length exoTNA (200 bp) contained detectable tumor-derived nucleic acids in the exosome from a NSCLC patient’s plasma [11]. However, the high molecular weight DNA (10–15 kb long) was more abundant in the exosomes from pleural fluids in this study than it was from the plasma. Therefore, the target size of the exoNAs depends upon the clinical sample types, and should be considered in the detection of *EGFR* mutations in NSCLC patients.

One limitation of our study is that matched tissue sampling was not performed in most cases. This might be the reason for the lower frequency of T790M mutations in our study than the previously reported prevalence of 40–55% [29]. However, in 22 of 64 patients (34.4%) who progressed despite EGFR-TKI therapy, the T790M mutation was identified in one or more liquid biopsy platforms (using the plasma or pleural fluid). We identified differences in the analytical performances of each type of specimen and nucleic acid using data from these patients.

Previous reports suggested that exoNA could be a novel DNA source for genetic testing [30]. In our study, the distribution of nucleic acid from exosomes was different than that from a cell pellet or supernatant. The nucleic acid from exosomes was enriched, high molecular weight DNA. This finding was consistent with that from previous reports [13, 30]. Further studies with larger cohorts are needed to substantiate our findings.

## Conclusion

In conclusion, combined TNA from the plasma and exoTNA from the pleural fluid could be used as a feasible target for low-abundant *EGFR* mutant copies in NSCLC. We demonstrated the optimal analytical method to improve the sensitivity of detecting *EGFR* mutations in different types of clinical samples.

## Supplementary Information

Additional file 1: **Fig. S1.** Nucleic acid yield plots from exoTNA and cfDNA; **Fig. S2.** Assessment of T790M mutant alleles according to different sources of tumor-derived nucleic acids (exosomes, cell pellet and supernatant) from the pleural fluid and extraction methods; **Fig. S3.** The distribution of isolated nucleic acids; **Table S1.** List of genes included in Oncomine Pan-Cancer Cell-Free Assay; **Table S2.** Clinical characteristics and plasma mutation test results from 54 patients; **Table S3.** Clinical characteristics and *EGFR* mutation results identified in the pleural fluid from 13 patients.

## Data Availability

The datasets supporting the conclusions of this article are included within the article and its additional files.

## Abbreviations

EGFR: Epidermal growth factor receptor
NSCLC: Non-small-cell lung cancer
TKIs: Tyrosine kinase inhibitors
ctDNA: Circulating tumor DNA
exoNA: Exosomal nucleic acid
exoTNA: Exosomal total nucleic acid
cfDNA: Cell-free DNA
BAL: Bronchoalveolar lavage
ddPCR: Droplet digital PCR
NAs: Nucleic acids
LOD: Limit of detection
NGS: Next-generation sequencing
