# Supplementary material for: Exosome-based detection of EGFR T790M in plasma and pleural fluid of prospectively enrolled non-small cell lung cancer patients after first-line tyrosine kinase inhibitor therapy
Source: Cancer Cell Int. 2021 Jan 12;21:50. doi: 10.1186/s12935-021-01761-x (PMC7802208; doi:10.1186/s12935-021-01761-x)
Supplement: Supplementary file 1 — Additional file 1: Fig. S1. Nucleic acid yield plots from exoTNA and cfDNA; Fig. S2. Assessment of T790M mutant alleles according to different sources of tumor-derived nucleic acids (exosomes, cell pellet and supernatant) from the pleural fluid and extraction methods; Fig. S3. The distribution of isolated nucleic acids; Table S1. List of genes included in Oncomine Pan-Cancer Cell-Free Assay; Table S2. Clinical characteristics and plasma mutation test results from 54 patients; Table S3. Clinical characteristics and EGFR mutation results identified in the pleural fluid from 13 patients. [file 12935_2021_1761_MOESM1_ESM.docx]

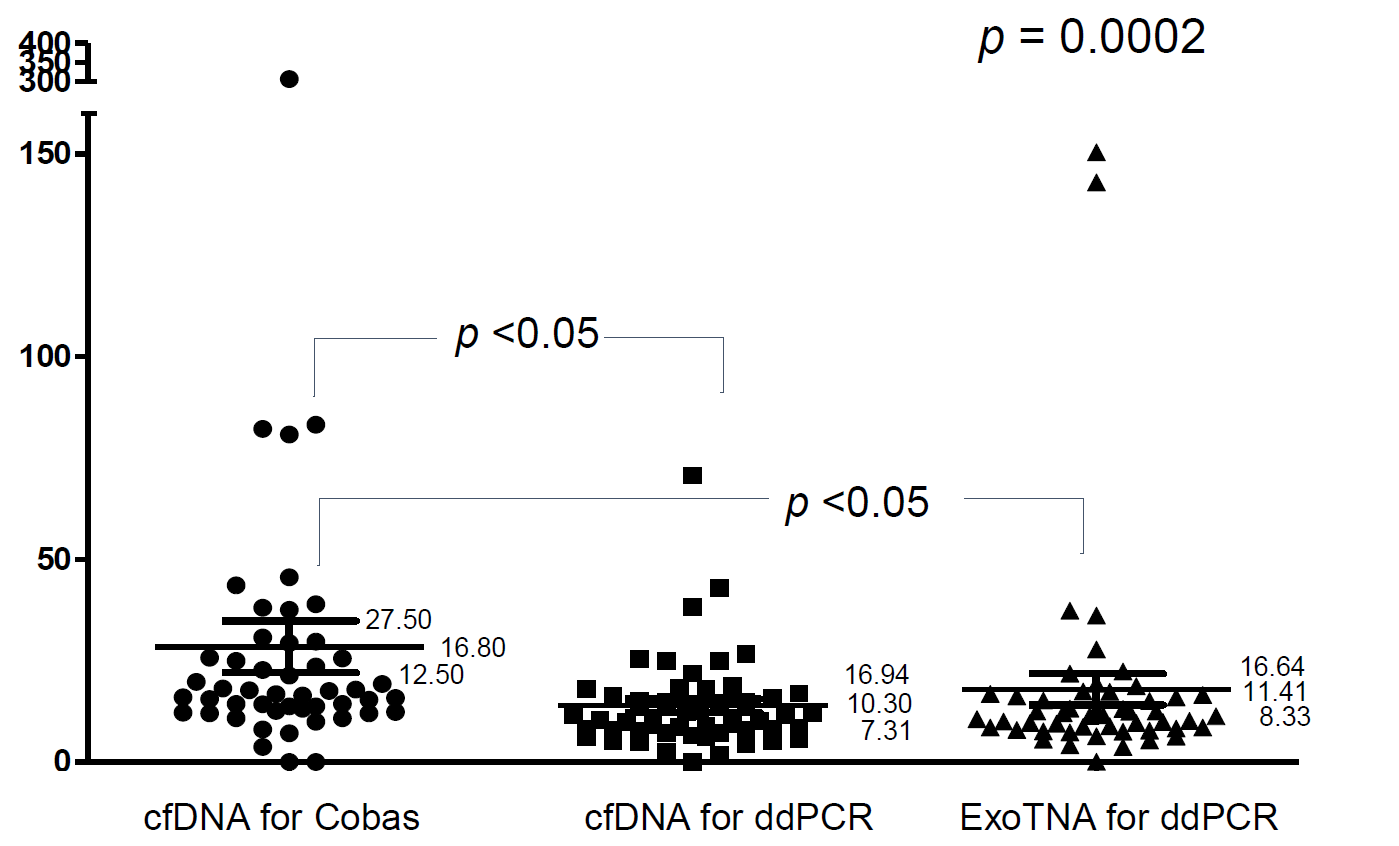


**Fig. S1.** Nucleic acid yield plots from exoTNA and cfDNA. The y-axis indicates the yield of nucleic acids in nanograms per sample, and the x-axis shows sample materials. Black horizontal bars indicate median with interquartile range.


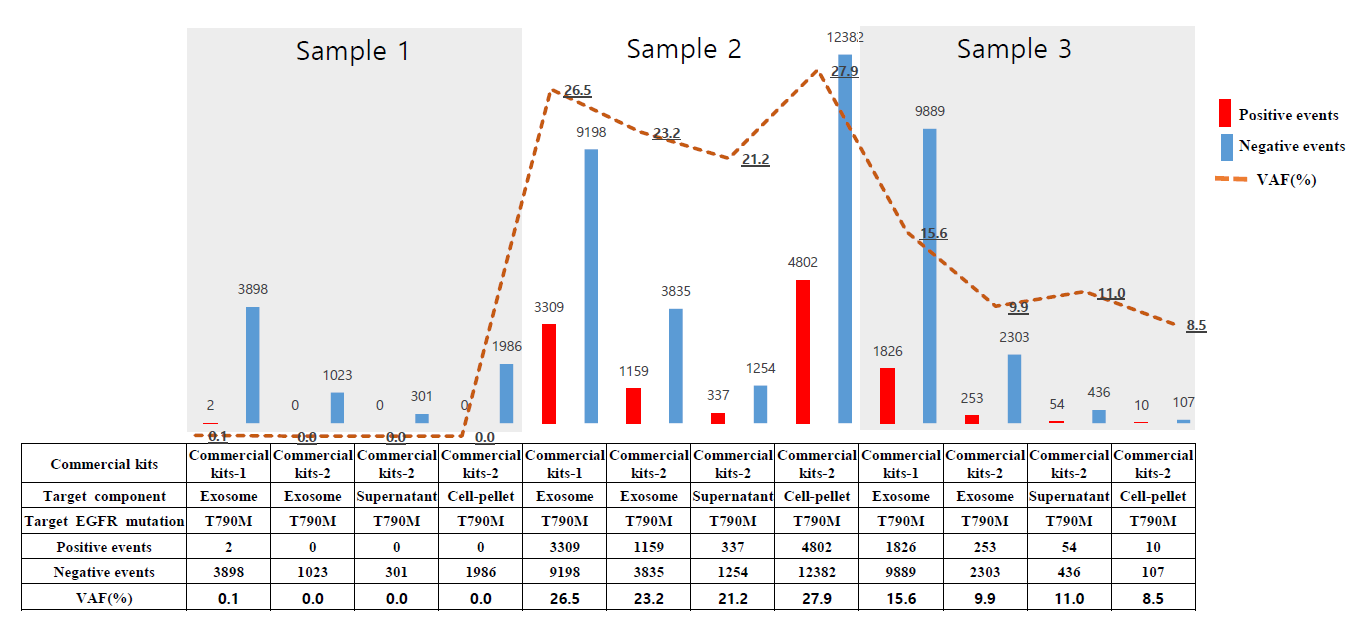


**Fig. S2.** Assessment of T790M mutant alleles according to different sources of tumor-derived nucleic acids (exosomes, cell pellet, and supernatant) from pleural fluid and extraction methods. ExoTNA and DNA (cell pellet and supernatant) were extracted using different commercial kits. And T790M mutant alleles were assessed using ddPCR assay. Commercial kits 1, MagMAX™ Total Nucleic Acid Isolation Kit; Commercial kits 2, QIAamp DNA Mini Kit.


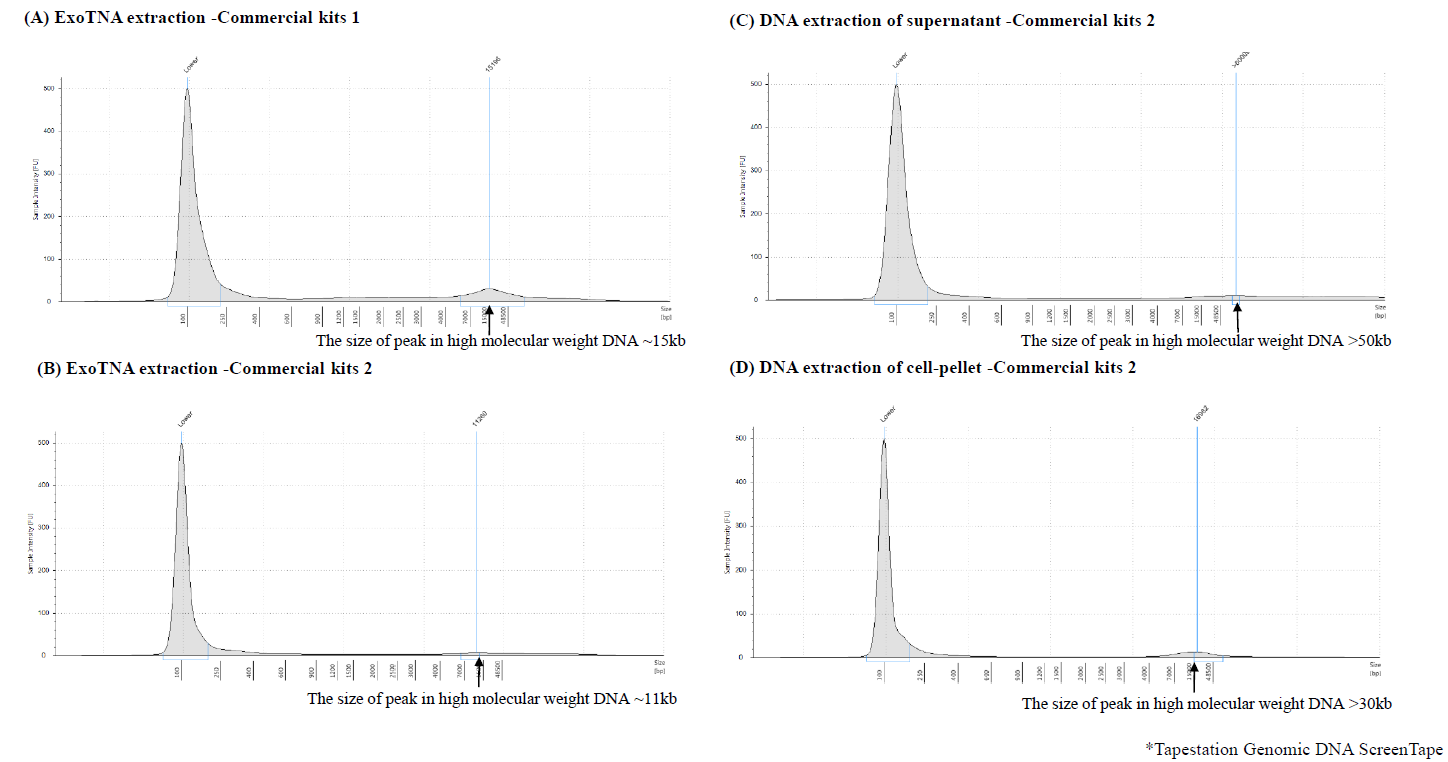


**Fig. S3.** The distribution of isolated nucleic acids. The high molecular weight DNA (10~15kb long) were more abundant in exosomes from pleural fluids. **(A)** ExoTNA was extracted using a Commercial kit 1 (MagMAX™ Total Nucleic Acid Isolation Kit), **(B)** ExoTNA was extracted using a Commercial kit 2 (QIAamp DNA Mini Kit), **(C)** total DNA in the supernatant of pleural fluids were extracted using a Commercial kit 2 (QIAamp DNA Mini Kit), **(D)** total DNA in cell-pellet of pleural fluids was extracted using a Commercial kit 2 (QIAamp DNA Mini Kit).

| **Table S1.** List of genes included in Oncomine Pan-Cancer Cell-Free Assay |
| --- |
| *AKT1, ALK, APC, AR, ARAF, BRAF, CCND1, CCND2, CCND3, CDK4, CDK6, CHEK2, CTNNB1, DDR2, EGFR, ERBB2, ERBB3, ERG, ESR1, ETV1, FBXW7, FGFR1, FGFR2, FGFR3, FGFR4, FLT3, GNA11, GNAQ, GNAS, HRAS, IDH1, IDH2, KIT, KRAS, MAP2K1, MAP2K2, MET, MTOR, MYC, NRAS, NTRK1, NTRK3, PDGFRA, PIK3CA, PTEN, RAF1, RET, ROS1, SF3B1 SMAD4, SMO, TP53* |

**Table S2.** Clinical characteristics and plasma mutation test results from 54 patients

| ID | Sex | Age | Histologic type | Tumor stage | M category | Prior EGFR-TKI treatment | Tissue sampling time | Tissue *EGFR* genotyping | Plasma collection date with regard to TKI therapy | Cobas *EGFR* genotyping | ddPCR *EGFR* genotyping (numbers of positive droplets/wild type droplets) | *EGFR* mutation from NGS (VAF %) | Other mutation from NGS (VAF %) |
| --- | --- | --- | --- | --- | --- | --- | --- | --- | --- | --- | --- | --- | --- |
| P1 | F | 73 | ADC | IVa | M1a | Erlotinib | Initial | E20ins | Post 8 months | Not detected | Not detected | Not detected | Not detected |
| P2 | M | 61 | ADC | IVb | M1c | Erlotinib | Initial | L858R | Post 14 months | L858R | L858R (306/355) | L858R | Not detected |
| P3 | F | 54 | ADC | IVb | M1c | Afatinib | Initial | E19del | Post 26 months | E19del, T790M | E19del (190/380), T790M (64/620) | E19del (17.6), T790M (7.98) | *TP53* H193Y (0.7) |
| P4 | M | 59 | ADC | IVb | M1c | Erlotinib | Initial | E19del | Post 9 months | E19del | E19del (21/685) | E19del (0.6) | *TP53* L252del (0.3) |
| P5 | F | 67 | ADC | IVb | M1c | Afatinib | Initial | L858R | Post 15 months | L858R | L858R (52/374) | L858R (5.7) | *TP53* R196Q (0.1) |
| P6 | M | 74 | ADC | IVb | M1c | Gefitinib | Initial | L858R | Post 12 months | E19del, L858R | L858R (34/557), T790M (6/1058) | Not done | Not done |
| P7 | F | 59 | ADC | IVb | M1c | Afatinib | Initial | L858R | Post 13 months | L858R | L858R (11/210) | Not done | Not done |
| P8 | F | 79 | ADC | IVa | M1b | Gefitinib | Initial | L858R | Post 13 months | E19del, L858R | L858R (285/395) | L858R (26.7) | *TP53* R175H (0.2), *EGFR* amp |
| P9 | M | 55 | ADC | IVa | M1a | Afatinib | Initial | E19del | Post 25 months | E19del, T790M | E19del (11/342), T790M (3/485) | E19del (1.6), T790M (2.4) | Not detected |
| P10 | F | 52 | ADC | IVa | M1a | Gefitinib | Initial | E19del | Post 17 months | E19del, T790M | E19del (44/337), T790M (7/470) | E19del (2.1), T790M (0.9) | Not detected |
| P11 | F | 55 | ADC | IVb | M1c | Erlotinib | Initial | E19del | Post 10 months | E19del, T790M | E19del (208/550), T790M (22/834) | E19del (12.0), T790M (1.2) | Not detected |
| P12 | F | 68 | ADC | IVa | M1a | Gefitinib | Initial | E19del | Post 17 months | Not detected | Not detected | Not done | Not done |
| P13 | F | 76 | ADC | IVb | M1b | Afatinib | Initial | E19del | Post 28 months | E19del | E19del (2/810) | Not done | Not done |
| P14 | F | 70 | ADC | IVa | M1a | Erlotinib | Initial | E19del | Post 24 months | Not detected | Not detected | Not done | Not done |
| P15 | M | 57 | SCC | IVb | M1c | Afatinib | Initial | L861Q | Post 24 months | Not detected | Not detected | Not done | Not done |
| P16 | M | 77 | ADC | IVa | M1b | Gefitinib | Initial | L858R | Post 7 months | L858R | L858R (7/800) | Not done | Not done |
| P17 | F | 83 | ADC | IVb | M1c | Erlotinib | Initial | E19del | Post 13 months | Not detected | Not detected | Not detected | Not detected |
| P18 | F | 61 | ADC | IVb | M1b | Gefitinib | Initial | L858R | Post 21 months | Not detected | Not detected | Not done | Not done |
| P19 | F | 40 | ADC | IVa | M1a | Gefitinib | Matched with plasma | E19del, T790M | Post 10 years | E19del, T790M | E19del (235/651), T790M (3/895) | E19del (11.8), T790M (1.0) | *CDK6* amp, *KRAS* amp, *CCND1* amp |
| P20 | F | 53 | ADC | IVa | M1a | Gefitinib | Initial | E19del | Post 16 months | E19del | E19del (6/389), T790M (5/578) | T790M (0.4) | *PIK3CA* E545G, *TP53* E56fs |
| P21 | F | 72 | ADC | IVb | M1c | Gefitinib | Initial | L858R | Post 12 months | L858R | L858R (4/394) | Not done | Not done |
| P22 | M | 72 | ADC | IVb | M1c | Erlotinib | Initial | L858R | Post 25 months | L858R, T790M | L858R (208/208), T790M (215/273) | L858R (47.0), T790M (47.7) | *TP53* V172F (1.9), *CTNNB1* I35N (0.3), *EGFR* amp, *CDK6* amp |
| P23 | F | 60 | ADC | IVb | M1c | Erlotinib | Initial | E19del | Post 6 years | E19del, T790M | E19del (5056/4644), T790M (2098/4943) | E19del (29.1), T790M (24.1) | *TP53* S241C (17.9), *EGFR* amp |
| P24 | F | 48 | ADC | IVb | M1b | Gefitinib | Initial | E19del | Post 13 months | Not detected | Not detected | Not detected | Not detected |
| P25 | F | 75 | ADC | IVa | M1a | Gefitinib | Initial | L858R | Post 18 months | L858R | L858R (2/234) | Not done | Not done |
| P26 | F | 74 | ADC | IVa | M1a | Afatinib, Gefitinib | Initial | E19del | Post 37 months | E19del, T790M | E19del (35/830), T790M (13/1096) | E19del (3.6), T790M (1.4) | *TP53* V274L (1.8), *TP53* R156G (0.54) |
| P27 | F | 83 | ADC | IVb | M1c | Gefitinib | Initial | E19del | Post 24 months | E19del | E19del (5/479) | E19del (0.4), T790M (0.5) | *MAP2K1* F53L (0.2), *TP53* c.920-1G>T (0.728), *TP53* T102fs (0.1), *FGFR3* K715I (0.1) |
| P28 | F | 69 | ADC | IVb | M1c | Afatinib | Initial | E19del | Post 8 months | E19del | E19del (2/363) | E19del (0.3) | *KRAS* G12D (0.1) |
| P29 | F | 65 | ADC | IVa | M1a | Afatinib | Initial | E19del | Post 21 months | Not detected | Not detected | E19del (0.4) | *FBXW7* R479Q (0.1), *TP53* A84fs (0.1) |
| P30 | F | 61 | ADC | IVb | M1b | Gefitinib | Initial | L858R | Post 25 months | L858R | L858R (2/431) | L858R (0.3) | *PIK3CA* E545K (5.1), *TP53* Q144* (0.3), *MAP2K2* E60L (0.4), *TP53* Q100fs (0.1) |
| P31 | F | 75 | ADC | IVb | M1c | Erlotinib | Initial | L858R | Post 10 months | Not detected | Not detected | Not detected | Not detected |
| P32 | M | 70 | ADC | IVb | M1c | Gefitinib | Initial | L858R | Post 11 months | L858R, T790M | L858R (201/5388), T790M (107/4453) | L858R (4.0), T790M (3.9) | Not detected |
| P33 | M | 56 | ADC | IVb | M1c | Gefitinib | Matched with plasma | E19del | Post 10 years | E19del | E19del (2337/3691) | E19del (26.0) | *PIK3CA* E545K (1.2), *TP53* M231I (0.1), *TP53* R213* (28.7) |
| P34 | F | 85 | ADC | IVb | M1c | Gefitinib | Initial | E19del | Post 12 months | E19del | E19del (2/178) | Not done | Not done |
| P35 | M | 52 | ADC | IIa | M0 | Gefitinib | Initial | L858R | Post 5 years | Not detected | Not detected | Not done | Not done |
| P36 | M | 70 | ADC | IVa | M1b | Afatinib | Initial | L858R | Post 17 months | L858R | L858R (3/913) | L858R (0.5) | Not detected |
| P37 | F | 55 | ADC | IVa | M1a | Gefitinib | Initial | E19del | Post 13 months | E19del | E19del (95/875) | E19del (8.1) | *PIK3CA* E545K (14.8), *TP53* S241C (8.8) |
| P38 | M | 56 | ADC | IVb | M1c | Afatinib, Gefitinib | Initial | E19del | Post 31 months | E19del, T790M | E19del (257/544), T790M (47/485) | E19del (31.8), T790M (11.0) | *TP53* P278T (2.4), *APC* S1407* (0.1) |
| P39 | M | 61 | ADC | IVa | M1a | Afatinib | Initial | E19del | Post 16 months | E19del | E19del (206/2595) | E19del (4.7) | *KRAS* G13D (0.7), *KRAS* G12D (0.2) |
| P40 | F | 77 | ADC | IVa | M1a | Gefitinib | Matched with plasma | E19del, T790M | Post 22 months | Not detected | Not detected | Not detected | *GNAS* R201C (0.2) |
| P41 | F | 84 | ADC | IVa | M1a | Gefitinib | Initial | L858R | Post 18 months | L858R | L858R (15/1120) | L858R (1.7) | *HRAS* G12V (0.1), *TP53* Y103fs (0.5), *TP53* C176F (0.1) |
| P42 | F | 63 | ADC | IVa | M1b | Afatinib | Initial | L858R | Post 4 months | L858R | L858R (2/245) | Not done | Not done |
| P43 | F | 63 | ADC | IVa | M1b | Gefitinib | Initial | E19del | Post 17.8 months | E19del | E19del (13/1239), T790M (5/742) | E19del (0.7), T790M (1.2) | *PIK3CA* R88Q (0.3), *TP53* Y220C (0.2) |
| P44 | F | 70 | ADC | IIIb | M0 | Erlotinib | Initial | E19del | Post 40 months | E19del | E19del (3/1071) | E19del (0.5) | *TP53* D281E (0.6), *TP53* R273H (0.5), *SMAD4* A118V (0.3), *TP53* A84fs (0.1) |
| P45 | F | 62 | ADC | IVa | M1b | Gefitinib | Initial | L858R | Post 36 months | L858R | L858R (200/2344) | L858R (9.0) | *APC* T1556fs (0.5) |
| P46 | F | 73 | ADC | Iva | M1c | Afatinib | Initial | E19del | Post 6 months | Not detected | Not detected | Not detected | Not done |
| P47 | F | 70 | ADC | IVa | M1b | Gefitinib | Unknown | Unknown | Post 8 years | Not detected | Not detected | Not detected | *GNAS* R201H (0.2), *TP53* V143M (0.4) |
| P48 | F | 77 | ADC | IVb | M1c | Afatinib | Initial | E19del | Post 10 months | E19del | E19del (8/1351) | E19del (0.4), L858R (0.2) | *CTNNB1* S45P (0.3), *ERBB3* E928G (0.3) |
| P49 | M | 69 | ADC | IVa | M1b | Afatinib | Initial | E19del | Post 14 months | E19del | E19del (2/688) | Not done | Not done |
| P50 | F | 56 | ADC | IVa | M1b | Erlotinib | Initial | L858R | Post 25 months | L858R, T790M | L858R (51/679), T790M (23/752) | E19del (0.1), L858R (5.5), T790M (3.0) | *BRAF* Y472C (0.4), *ERBB3* E928G (0.5), *TP53* S303N (2.6), *TP53* Q331* (0.3), *TP53* R213* (0.2), *SMAD4* R361C (0.2) |
| P51 | F | 63 | ADC | IVa | M1b | Gefitinib | Initial | E19del | Post 20 months | E19del | E19del (11/1613), T790M (3/888) | Not done | Not done |
| P52 | F | 63 | ADC | IVb | M1c | Gefitinib, Erlotinib | Initial | E19del | Post 27 months | E19del | E19del (523/3833) | E19del | *APC* T1556fs (0.4), *EGFR* amp, *CDK4* amp |
| P53 | M | 61 | ADC | IVa | M1a | Afatinib | Initial | E19del | Post 25 months | E19del | E19del (45/1058) | E19del | Not detected |
| P54 | F | 73 | ADC | IVa | M1a | Gefitinib | Initial | E19del | Post 21 months | Not detected | E19del (2/827) | Not done | Not done |
| VAF, variant allele frequency; ADC, adenocarcinoma; SCC, squamous cell carcinoma; E19del, exon 19 deletion; amp, amplification. | | | | | | | | | | | | | |

**Table S3.** Clinical characteristics and *EGFR* mutation results identified in pleural fluid from 13 patients

| ID | Sex | Age | Histologic type | Tumor stage | M category | Prior EGFR-TKI treatment | Tissue sampling time | Tissue *EGFR* genotypoing | Pleural fluid collection with regard to TKI therapy | Plasma *EGFR* result (mutation detection method) | ddPCR using exoTNA extracted from supernatants (numbers of positive droplets/wild type droplets) | ddPCR using total DNA extracted from cell pellet (numbers of positive droplets/wild type droplets) |
| --- | --- | --- | --- | --- | --- | --- | --- | --- | --- | --- | --- | --- |
| P23 | F | 60 | ADC | IVb | M1c | Erlotinib | Initial | E19del | Post 6 years | E19del, T790M (Cobas, ddPCR, and NGS) | E19del (6601/8193), T790M (3309/9198) | E19del (7603/10755), T790M (4802/12382) |
| P25 | F | 75 | ADC | IVa | M1a | Gefitinib | Initial | L858R | Post 18 months | L858R (Cobas and ddPCR) | L858R (9725/7641), T790M (3011/9345) | L858R (703/428), T790M (170/944) |
| P54 | F | 73 | ADC | IVa | M1a | Gefitinib | Initial | E19del | Post 21 months | E19del (Cobas and ddPCR) | E19del (134/1444) | E19del (3/720) |
| P55 | F | 49 | ADC | IVa | M1b | Afatinib | Initial | E19del | Post 10 months | E19del (Cobas) | E19del (14/427) | E19del (4/1144) |
| P56 | F | 81 | ADC | IVa | M1a | Gefitinib | Initial | L858R | Post 6 months | Not done | L858R (1182/3662), T790M (4/4591) | L858R (13044/17268) |
| P57 | F | 43 | ADC | IVa | M1a | Gefitinib | Initial | E19del | Post 16 months | Not done | E19del (8584/7982), T790M (5958/8438) | E19del (12288/13717), T790M (1187/17088) |
| P58 | F | 76 | ADC | IVb | M1c | Gefitinib | Initial | Not done | Post 22 months | Not done | L858R (294/1107), T790M (77/1635) | L858R (11422/11665), T790M (594/14216) |
| P59 | F | 77 | ADC | IVa | M1a | Gefitinib | Initial | E19del | Post 11 months | Not done | E19del (155/145) | E19del (11377/14888) |
| P60 | M | 51 | SCC | IVa | M1a | Gefitinib | Initial | Not done | Post 9 months | Not done | Not detected | Not detected |
| P61 | F | 81 | ADC | IVa | M1a | Gefitinib | Initial | L858R | Post 13 months | Not done | L858R (215/3300), T790M (2/3898) | L858R (57/1511) |
| P62 | M | 60 | ADC | IVa | M1a | Gefitinib | Initial | G719X, S768I | Post 36 months | Not done | Not detected | Not detected |
| P63 | F | 41 | ADC | IVa | M1a | Gefitinib | Initial | L858R | Post 12 months | Not done | L858R (10378/7076), T790M (1826/9889) | L858R (61/53), T790M (10/107) |
| P64 | F | 75 | ADC | IVa | M1a | Gefitinib | Initial | L858R | Post 16 months | Not done | L858R (461/13317) | L858R (18/1405) |
| ADC, adenocarcinoma; SCC, squamous cell carcinoma; E19del, exon 19 deletion. | | | | | | | | | | | | |
